# Available Treatments and Adjunctive Therapies for Polycystic Ovarian Syndrome (PCOS) Patients of Reproductive Age: A Scoping Review

**DOI:** 10.7759/cureus.70501

**Published:** 2024-09-30

**Authors:** Lisa Cochran, Riley Nadolny, Kristen Garcia, Kimberly A Kluglein, Alexis Yagoda, Prachi Gandhi, Jordan Dressel, Barbara Prol, Ria Peralta, Arianne Shipp, Joshua M Costin

**Affiliations:** 1 Dr. Kiran C. Patel College of Osteopathic Medicine, Nova Southeastern University, Fort Lauderdale, USA; 2 Department of Medical Education, Nova Southeastern University Dr. Kiran C. Patel College of Allopathic Medicine, Fort Lauderdale, USA

**Keywords:** hyperandrogenism, menstrual irregularities hormonal imbalance, ovarian cyst, polycystic ovary syndrome (pcos), systematic scoping review, treatment choices

## Abstract

Polycystic ovarian syndrome (PCOS) impacts the health of women worldwide. It is a condition consisting of dysfunctional cystic ovaries resulting in hormonal imbalance. Many women have symptoms such as infertility, increased production of androgens, and insulin resistance. Barriers to effective treatment of PCOS include issues such as delays in diagnosis and inconsistencies in treatment plans among physicians. Despite the current use of available medications to decrease symptomatology, women with PCOS continue to report a decreased quality of life.

Using the electronic databases PubMed, Cumulative Index to Nursing and Allied Health Literature (CINAHL), and ScienceDirect, a scoping review was conducted on the globally available treatments for PCOS. After applying pre-determined inclusion criteria, 41 studies were included in this scoping review. The literature on the available treatments for PCOS revealed a wide range of therapeutics with evidence of reduction of symptoms and/or improvement in fertility status and pathological processes such as insulin resistance, hormone imbalance, obesity, inflammation, and infertility. Dozens of treatment options for PCOS have been identified, including new medications and modifications to existing treatment regimens. The hormonal drug Fezolinetant demonstrated effective suppression of hyperandrogenism. Drugs used to treat diabetes, such as Liraglutide, were found effective for weight loss. Green cardamom, cinnamon, and other supplements proved effective in treating metabolic dysfunction. Alternative approaches, such as osteopathic manipulative therapy and acupuncture, decreased sympathetic tone and androgen levels.

This review provides a succinct overview of PCOS therapies that can be used by those with PCOS and their physicians everywhere. With a better understanding of their options, women with PCOS can become more involved in the decision-making process to improve their health. More research is needed on novel therapies that aim to reduce the primary pathogenesis of PCOS.

## Introduction and background

Polycystic ovarian syndrome (PCOS) is a highly prevalent endocrine disorder affecting reproductive-aged women [1]. The World Health Organization (WHO) states that over 116 million women are affected by PCOS [2]. Although the etiology of PCOS is unknown, it is believed to be due to a combination of environmental and genetic factors [3]. Despite the prevalence of PCOS, inconsistencies have been reported among physicians regarding diagnosis [4]. There is a wide range of reported symptoms and presentations of PCOS that vary between age groups, ethnicities, and high-risk populations, making it difficult to diagnose. This is particularly prevalent when patients present with only one hallmark of the disease, leading to an estimate that up to 70% of women with PCOS remain undiagnosed [2]. Furthermore, other endocrine disorders such as thyroid disease, Cushing's disease, hyperprolactinemia, and hypogonadism must be ruled out, making PCOS a diagnosis of exclusion [5]. This can lead to delays in diagnosis and frustration among patients. Up to one-third of patients reported a delay of over two years before receiving a diagnosis of PCOS, and 40% reported visiting three or more physicians in the process [6]. Even after a successful diagnosis, PCOS patients often report a lack of understanding of the diagnosis and of the treatments available to them [7]. 

The exact cause of PCOS is still unknown, but it is believed to be a multifactorial disease. Genetic abnormalities involved in steroid production have been linked to PCOS. It is thought that environmental factors, such as obesity or insulin resistance, may interact with these genetic predispositions, contributing to the onset or progression of the condition. The underlying mechanism of PCOS is driven by a state of excess androgens. Most PCOS patients present with functional ovarian hyperandrogenism (FOH), which is characterized by an exaggerated release of 17-hydroxyprogesterone in response to gonadotropins. A potential underlying mechanism of FOH is thought to involve excess insulin, which disrupts the ovaries' responsiveness to luteinizing hormone (LH), leading to dysregulation of the ovulatory cycle. Elevated LH levels can worsen PCOS symptoms by increasing the production of androgens. Excess androgens and insulin can cause over-recruitment of primordial follicles and premature development of granulosa cells, which increases their estrogen production [5]. 

Dysregulation of the ovaries can disrupt the pulsatile release of gonadotropin-releasing hormone (GnRH), which can explain the elevated LH to follicle-stimulating hormone (FSH) ratio (LH:FSH) commonly seen in PCOS patients. The relative deficiency in FSH may hinder the conversion of androgen to estradiol in the granulosa cells due to decreased aromatase activity. Consequently, serum androgen levels rise and are converted to estrogen by peripheral fat tissue, a process that is hypothesized to be intensified in obese patients. This creates abnormal sustained negative feedback on the hypothalamus that worsens the cyclic nature of this disease [5]. 

**Figure 1:**
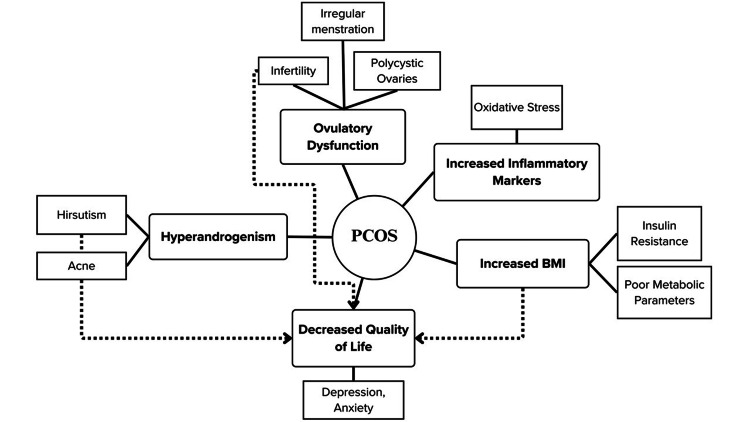
Common symptoms of polycystic ovarian syndrome (PCOS) Patients with PCOS may experience any or all of a constellation of symptoms related to increased body mass index (BMI), increased inflammatory markers, hyperandrogenism, ovulatory dysfunction causing menstrual irregularities, and decreased quality of life (QoL). Image credit: Lisa Cochran

PCOS is diagnosed by the presence of at least two of three characteristic symptoms: ovarian cysts, high levels of serum androgens (hyperandrogenism), and irregular or absent menstruation (Figure 1) - known as the Rotterdam criteria. Elevation of the ratio of LH to FSH (LH:FSH), hormones from the brain that act on the ovaries, is another sign of PCOS, although not required for diagnosis [5]. An imbalance of this ratio causes dysregulation or absence of ovulation in patients with PCOS. Although the diagnostic criteria outline three of the major symptoms of PCOS, many patients experience symptoms of PCOS caused by a dysregulation of hormones beyond the reproductive system, such as obesity, heart disease, infertility, type 2 diabetes, and/or depression (Figure 1) [8]. Additionally, because of a hormonal imbalance and insulin resistance, serum lipids may also be affected, leading to low amounts of high-density lipoprotein (HDL), high amounts of low-density lipoprotein (LDL), and high amounts of triglycerides in the blood, a lipid profile that poses a high risk for cardiovascular disease [9]. Many patients experience a decrease in their quality of life due to these constellations of symptoms (Figure 1), and the associated risk factors for increased morbidity and mortality suggest the need to close the gaps in treatment plans of women with PCOS. 

PCOS is currently incurable, so medications and lifestyle modifications aim to help alleviate the associated symptomatology [2]. Though there are no current therapies that address all PCOS-associated symptomatology at once, there are therapies that target individual symptoms [10]. This includes Metformin, commonly used in type 2 diabetes to improve insulin resistance [11], and oral contraceptive pills (OCPs) to help regulate the menstrual cycle [12]. If a patient desires fertility, the current first-line therapy is Clomiphene Citrate (CC), a hormone modulator that is used to promote ovulation in patients with ovulatory dysfunction [13,14]. Other treatments address the symptoms of hyperandrogenism seen in PCOS, such as hirsutism and acne, by decreasing the serum level of androgens [15]. An optimal effect may also require a decrease in body fat to prevent the conversion of estrogens to androgens by adipose tissue [10]. Using some treatments together may not be practical, as fertility treatment involves stimulating ovulation, which requires the carefully timed fluctuation of sex hormones, including estrogen. With the inevitable conversion of estrogen to androgen in the body, hirsutism may worsen while undergoing fertility treatments [16]. The complexity of these treatment regimens further perpetuates the barriers many women with PCOS face for maximum effectiveness of treatment. Moreover, the diversity of symptoms associated with PCOS forces treatment plans to be individualized, as there is no one-size-fits-all approach. Treatment is tailored to each patient's symptoms and fertility goals [17]. Substantial progress in research over the past few decades has contributed to our current knowledge of the evaluation and treatment of PCOS [18]. This review's objective is to improve the quality of life (QoL) in women living with PCOS by providing a condensed overview of all currently available evidence-based medications and therapies.

## Review

Methods

This review followed the Joanna Briggs Institute (JBI) guidelines for scoping reviews and utilized the Preferred Reporting Items for Systematic Reviews and Meta-Analyses (PRISMA) method [19]. All criteria were determined using the Population, Concept, and Context (PCC) framework before any search. Specifically, the population of interest were women aged 12-51 years diagnosed with PCOS (International Classification of Diseases, 10th Revision (ICD-10) E28.2). The concept was primary studies on the use and efficacy of treatments for PCOS. The context was articles published after 2014, written in the English language. Articles were additionally excluded if they were literature or scoping reviews or if a full text could not be obtained. 

Search Strategy

The first author, with assistance from the eighth and ninth authors, created the search terms, which were constructed from an analysis of key terms in Medical Subject Headings (MeSH). These search terms were utilized in each of the following databases on October 5, 2022: ScienceDirect, PubMed, and Cumulative Index to Nursing and Allied Health Literature (CINAHL). Final search terms included “Treatments AND PCOS” OR “Therapies and PCOS” OR “Management AND PCOS.”

Additional filters were applied to each database. In ScienceDirect, the following filters were used: publication title - Reproductive BioMedicine Online, Biomedicine and Pharmacotherapy, American Journal of Obstetrics and Gynecology; subject areas - Genetics and Molecular Biology, Medicine and Dentistry, Biochemistry; access type - open access and open archive. The following filters were used in PubMed: article type - books and documents, clinical trial, and randomized controlled trial. In CINAHL, the following filters were used: major heading contains Polycystic Ovarian Syndrome; publication: Journal of Endocrinology and Metabolism. Only free full text and abstracts, with unrestricted access and open archives, were included for all databases.

The final article selection was based on a two-step process, Tier 1 and Tier 2 reviews, using the Rayyan software (Rayyan Systems Inc., Cambridge, MA, www.rayyan.ai). For the Tier 1 review, all 10 authors reviewed the title and abstracts of the remaining studies, and consensus for inclusion was reached when at least eight out of 10 authors decided the study should be included. For the Tier 2 review, the first and second authors read the remaining full-text articles to ensure that each study met the inclusion criteria. The selection process was documented using a PRISMA flow chart.

Data Charting Process

A Microsoft Excel spreadsheet (Microsoft Corp., Redmond, WA) was used to chart relevant information from chosen final articles. Two reviewers charted the data independently. Following independent charting, inconsistencies were discussed among the two reviewers and a third to aid in the dispute and resolved based on the majority agreement.

Critical Appraisal of Individual Sources of Evidence (Quality Assessment)

To conduct the critical appraisal of the evidence, the JBI Critical Appraisal Checklist for Randomized Controlled Trials was utilized to identify potential bias [20]. The JBI Critical Appraisal Checklist enabled the classification of Tier 2 articles as having high, moderate, or minimal risk of bias.

Studies not classified as randomized controlled trials or that had no true control group were classified as high risk of bias. Studies with population groups that were too narrow, duplicates, or determined to be inconclusive were classified as moderate risk for bias. All studies at moderate or high risk for bias were excluded, and only studies at minimal risk were included in our analysis to maximize the study's validity.

Results

**Figure 2:**
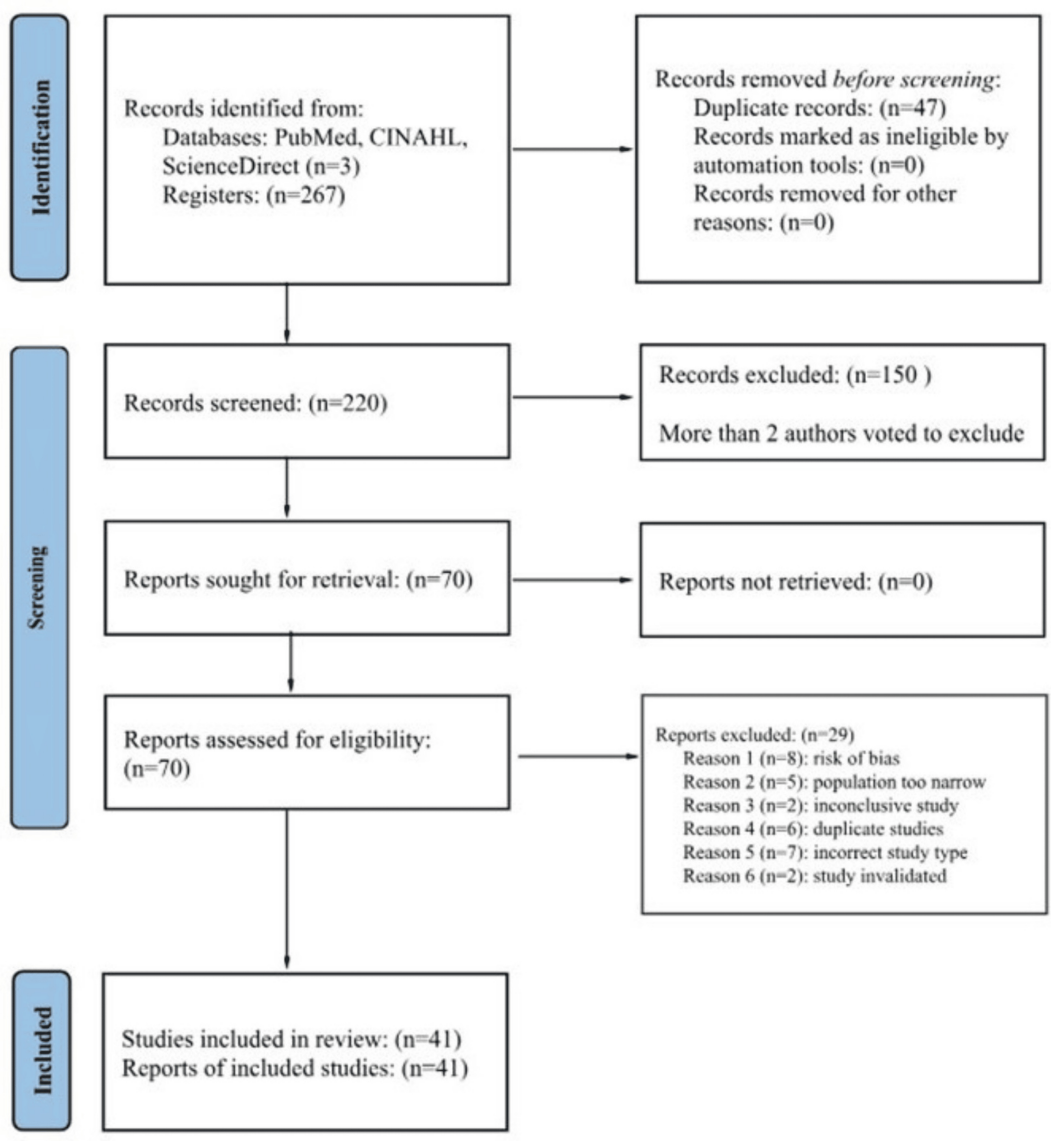
PRISMA flow diagram The PRISMA flow diagram tracks the process of study identification and selection. PRISMA, Preferred Reporting Items for Systematic Reviews and Meta-Analyses

**Figure 3:**
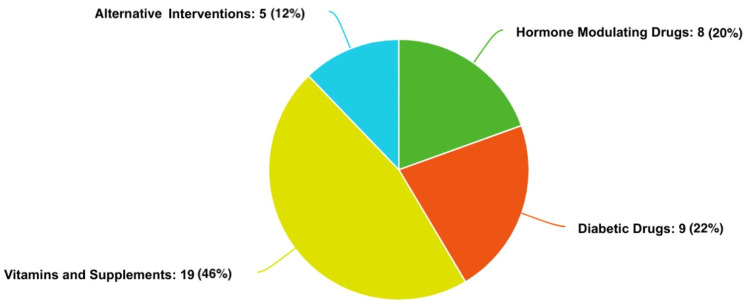
Relative proportion of studies in each treatment category Eight studies on hormone-modulating drugs make up 20% of the studies [21-28]. Nine studies on diabetic drugs make up 22% of the studies [29-37]. Nineteen studies on vitamins and supplements make up 46% of the studies [38-56]. Five studies on alternative interventions make up 12% of the studies [57-61]. For each treatment category, the design, data, conclusion, and limitations of each study are shown in Tables 2-5.

Searching the identified databases resulted in 267 articles, 47 of which were duplicates and were removed before screening. The remaining 220 articles were screened utilizing the predetermined inclusion and exclusion criteria, leaving 41 articles to be included in this review (Figure 2). The study types included are 39 randomized controlled trials, one non-randomized controlled trial, and one prospective cohort study. The geographical span of all studies included in this review is worldwide, with the majority occurring in Iran (n = 12), China (n = 8), and the United States (n = 7). The types of PCOS treatments discussed in the included studies consisted of hormone drugs (20%), diabetic drugs (22%), vitamins and supplements (46%), and alternative interventions (12%) (Table 1 and Figure 3).

**Table 1:** Characteristics of Different Treatments According to Symptomatology The types of polycystic ovarian syndrome (PCOS) treatments discussed in the studies included eight hormone-modulating drugs, nine diabetes drugs, 19 vitamins and supplements, and five alternative interventions. The symptomatology targeted by these treatments included anthropometric measures, including insulin resistance, hyperandrogenism, inflammatory markers, fertility, QoL, and metabolic parameters. CBT = cognitive behavioral therapy; COC = combined oral contraceptive; GnRH = gonadotropin releasing hormone; HMG = human menopausal gonadotropin; IU = international units; OC = oral contraceptive; OCP = oral contraceptive pill; QoL = quality of life

Treatment Type	Decreased Hyperandrogenism	Improved Metabolic Parameters	Improved Insulin Resistance/Sensitivity	Decreased Serum Inflammatory Markers	Improved Fertility/Pregnancy Outcomes	Improved QoL/Mental Health	Improved Anthropometric Measurements
Hormone-Modulating Drugs	Fezolinetant [21]	Ethinyl estradiol (OC) ± Metformin [22]; OCP + Lifestyle Modification [23]; Drospirenone-Containing COC [24]	Ethinyl estradiol (OC) ± Metformin [22]	N/A	Letrozole [25]; GnRH Antagonist [26]; GH [27]; Letrozole + HMG [28]	N/A	N/A
Diabetes Drugs	Liraglutide [29]; Metformin [30]; Pioglitazone + Metformin Complex [31]	Liraglutide [29,32]	Sitagliptin [33]; Exenatide + Metformin [34]; Pioglitazone [35]	Metformin [36]	N/A	Metformin [30]; Pioglitazone + Metformin Complex [31]	Exenatide + Metformin [37]; Liraglutide [29,32]
Vitamins and Supplements	Dextrin Prebiotic [38]; Omega-3 + Coenzyme Q10 [39] Puerarin [40]; Alpha-Lactalbumin + Myo-Inositol [41]; 4000 IU Vitamin D [42]; Selenium + Probiotic [43] Vitamin D + Probiotic [44]	Puerarin [40]; 4000 IU Vitamin D [42]; Selenium + Probiotic [43]; Vitamin D + Probiotic [44]; Brown-Milled Flaxseed [45]; Cinnamon [46]; Vitamin D [47]	Alpha-Lactalbumin + Myo-Inositol [41]; Vitamin D + Probiotic [44]; Brown-Milled Flaxseed [45]; Thylakoid-Rich Spinach Extract [47]; Vitamin D [48,49]	Brown-Milled Flaxseed [45]; 4000 IU Vitamin D [42]; Selenium + Probiotic [43]; Vitamin D + Probiotic [44]; Green Cardamom [50]; Resveratrol [51]; Synbiotic [52]	Alpha-Lactalbumin + Myo-Inositol [41]; Herbal Medicine [53]; Letrozole + Lactofem [54]	Vitamin D + Probiotic [44]; Selenium + Probiotic [43]; Herbal Medicine [53]; Letrozole + Lactofem [54]; Menaquinone-7 (Vitamin K2) [55]; Magnesium [56]	N/A
Alternative Interventions	Phlebotomy [57]; Osteopathic Manipulative Treatment [58]	Phlebotomy [57]	Phlebotomy [57]	N/A	Acupuncture + Low Frequency Electrical Stimulation [59]	Structured Health Education [60]; CBT + Lifestyle Modifications [61]	CBT + Lifestyle Modifications [61]

Hormone-Modulating Drugs

Hormone therapies are used for both the management of menstrual symptoms and the treatment of infertility (Table 2) and represent 20% of the included studies (Figure 3). Eight studies tested pharmaceuticals that target hormones and evaluated their effects in women with PCOS. Of the studies investigated, three studied the effects of OCPs. These three studies on OCPs showed improvement in metabolic profiles, such as lowering adiponectin [22], raising HDL levels [23], and controlling blood pressure [24]. Another study investigated Fezolinetant, a neurokinin (NK)-receptor antagonist commonly used to treat symptoms of menopause, and found a significant reduction in hyperandrogenism and LH-to-FSH ratio [21]. The remaining studies aimed at improving fertility issues associated with PCOS. The studies investigating Letrozole, Cetrotide, growth hormone (GH) supplementation, and the gonadotropin hormone-releasing hormone (GnRH) antagonist showed improvements in fertility outcomes, including pregnancy rates, ovarian hyperstimulation syndrome (OHSS), and granulosa cell survival [25-28].

**Table 2:** Studies on Hormone-Modulating Therapies CC = clomiphene citrate; COC = combined oral contraceptive; HDL = high density lipoprotein; HMG = human menopausal gonadotropin; LDL = low density lipoprotein; OC = oral contraceptive; OCP = oral contraceptive pill; OHSS = ovarian hyperstimulation syndrome; PCOS = polycystic ovarian syndrome; RCT = randomized control trial

Reference	Region/Country	Study Design	Data Collection	Study Aim	Conclusion	Limitations
Fraser et al., 2021 [21]	Europe	Randomized Controlled Trial	Fezolinetant 60 mg: n = 23; Fezolinetant 180 mg: n = 23; Placebo: n = 27	Investigate the use of Fezolinetant, an NK-3 receptor antagonist, in women with PCOS.	Fezolinetant improved markers of hyperandrogenism by decreasing the testosterone and LH:FSH ratio.	Small sample size, short study duration, focus on biochemical markers, one month of OCP discontinuation.
Bodur et al., 2018 [22]	Taiwan	Randomized Controlled Trial	Drospirenone + Ethinyl Estradiol: n = 21; Drospirenone + Metformin: n = 20; Metformin: n = 20; Control: n = 17	Investigation on the impact of drospirenone/ethinyl estradiol (OC) vs. in combination with Metformin on metabolic markers in PCOS patients who are non-obese.	OC alone was shown to decrease serum markers of poor metabolism (adiponectin, hsCRP). Metformin, in addition to OC, did not provide any significant differences.	Small sample size, short follow-up period, and inconsistencies in the measurement and interpretation of laboratory data.
Dokras et al., 2017 [23]	USA	Randomized Controlled Trial	Oral Contraception: n = 33; Intensive Lifestyle Modification Program: n = 29; Combination Therapy: n = 25	Investigate the effects of OCPs on lipoprotein particles and HDL in obese PCOS patients.	In obese women with PCOS, OCPs can be used in conjunction with lifestyle modifications to increase HDL, but this does not decrease LDL levels.	Short duration of study, only used one type and dose of OCP with a progestins/ethinyl estradiol, secondary analysis of an RCT that was designed prior to assess the impact of the selected interventions.
Wang et al., 2016 [24]	China	Randomized Controlled Trial	CPA Intervention: n = 49; DRP Intervention n = 50	Investigate the effects of COCs with drospirenone vs. COCs with cyproterone acetate in PCOS patients. Both are in conjunction with lifestyle changes and Metformin.	COCs with drospirenone combined in conjunction with lifestyle changes and Metformin showed more improvement in blood pressure control and metabolic markers than COCs with cyproterone in PCOS patients.	Small sample size, short follow-up period.
Amer et al., 2017 [25]	Europe	Randomized Controlled Trial	Clomiphene Citrate: n = 74; Letrozole: n = 75	Study if Letrozole would improve the rate of pregnancy more than CC in PCOS women who are subfertile.	Letrozole showed more improvements in primary ovulation over CC in PCOS patients, which caused them to have quicker and higher amounts of pregnancy rates.	Exclusion of women with BMI > 35.
Eftekhar et al., 2018 [26]	Iran	Randomized Controlled Trial	GnRH Antagonist Pretreatment: n = 38; Control Group (Gonal-f 150 IU): n = 50	Investigate if pretreatment of a GnRH antagonist, such as Cetrotide, influenced outcomes of pregnancy in PCOS patients.	GnRH antagonist administration had significantly higher pregnancy rates and reduction of ovarian hyperstimulation syndrome compared to the control group.	Lack of statistical power analysis, small sample size, treatment requires three SC injections.
Gong et al., 2020 [27]	China	Randomized Controlled Trial	PCOS + GH: n = 30; PCOS Only: n = 31; Control (w/o PCOS): n = 32	Investigate the impact growth hormone has on granulosa cell oxidative stress in PCOS patients.	For PCOS patients, growth hormone showed improvement in restricting the granulosa cells' apoptosis by the PI3K-Alk pathway.	Small sample size, symptomatology not measured.
Xi et al., 2015 [28]	China	Randomized controlled Trial	Letrozole + HMG: n = 94; CC + HMG: n = 90; HMG Only: n = 71	Investigate the impact of letrozole + CC + HMG in patients with PCOS that are CC-resistant and face infertility.	Letrozole with CC and HMG provided improvements in fertility rates, reduction in OHSS, in CC-resistant women with PCOS.	Specific population, non-randomized design.

Diabetic Drugs

Nine studies explored the potential benefits of using diabetic drugs to treat the related secondary symptomatology of PCOS, including weight gain, increased body fat, and insulin insensitivity (Table 3), representing 22% of the included studies (Figure 3). Liraglutide was used as the treatment drug in two of these studies, both of which found the drug effective in weight loss [29,32]. Two studies found that Metformin helped increase endogenous insulin secretion, as well as concomitant improvements in acne, hair loss, infertility, and endothelial function [30,36]. One study found Sitagliptin decreased visceral body fat and maximal glucose response [33]. Two studies showed Pioglitazone to be effective in improving sex hormone levels and insulin insensitivity [31,35]. The remaining two studies found that diabetic therapies such as Exenatide or a combination of Metformin and Exenatide achieve better insulin sensitivity and a higher rate of remission of prediabetes compared to Metformin alone among women with PCOS [34,37]. 

**Table 3:** Studies on Diabetic Drugs COM = metformin combined with exenatide; DCI-IPG = d-chiro-inositol-inositol phosphoglycan; DPP4 = dipeptidyl peptase-4; GH = growth hormone; HRQOL = health-related quality of life; NLRP-3 = nucleotide-binding domain, leucine-rich-containing family, pyrin domain-containing-3; OGTT = oral glucose tolerance test; PCOS = polycystic ovarian syndrome; PM = pioglitazone-metformin complex; QOL = quality of life; VAT = visceral adiposity

Reference	Region/Country	Study Design	Data Collection	Study Aim	Conclusion	Limitations
Elkind-Hirsch et al., 2022 [29]	USA	Randomized Controlled Trial	Liraglutide: n = 55; Placebo: n = 27	Investigate the use of Liraglutide vs. a placebo for weight loss and improvement of hyperandrogenism in obese PCOS patients.	Liraglutide showed more improvement compared to the placebo, by reducing body weight, hyperandrogenism, and other metabolic markers in women with PCOS and obesity.	Absence of gold-standard measure of insulin resistance, high drop-out rate due to no weight loss results.
Ou et al., 2016 [30]	Taiwan	Prospective Cohort Study	Questionnaires were administered to each participant (n = 109) before, during, and after Metformin treatment	Investigate the HRQOL of Chinese women with PCOS on metformin treatment.	Metformin can raise the QOL in women with PCOS by decreasing psychological and physical distress such as unwanted or excessive hair growth, acne, and fertility problems.	Homogeneous population studied, no objective measures were included, no comparison to other PCOS treatments, participants were aware of the study.
Guo et al., 2020 [31]	China	Randomized Controlled Trial	Metformin Complex Preparations: n = 28; Metformin: n = 26; Placebo: n = 21	Study the use of pioglitazone metformin complex preparation (PM) in psychological distress of PCOS patients.	This study found that PM decreased psychological distress by correcting markers like testosterone and also inhibiting NLRP3 inflammasome.	Small sample size, high drop-out rate, safety in pregnancy not confirmed.
Kahal et al., 2015 [32]	United Kingdom	Randomized Controlled Trial	Liraglutide: n = 19; Control: n = 17	Investigate the effect of liraglutide on the risk of developing cardiovascular disease and/or obesity in young PCOS patients and compare it to controls (similar weight and age without PCOS).	Liraglutide impacted both the control group and those with PCOS, with improvement in platelet function and weight loss. Showing this medication can be used for those with or without PCOS.	High drop-out rate, absence of placebo-treated group.
Devin et al., 2020 [33]	USA	Crossover Randomized Controlled Trial	Subjects were assigned to treatment order (Sitagliptin or Placebo): n = 18;	Investigate whether inhibition of DPP4 improves glycemic index, sex hormones and vascular function in PCOS patients.	Sitagliptin decreased VAT and the max glucose response to OGTT. It also improved the pulse interval and half-life of GH but showed no effect on overnight GH levels.	Small sample size, homogeneous population (BMI > 25), could not standardize GH timing due to menstrual cycle irregularities.
Ma et al., 2021 [34]	China	Randomized Controlled Trial	Metformin + Exenatide (Once Weekly): n = 25; Metformin: n = 25	Determine if exenatide combined with Metformin (COM) has an impact on metabolism, body weight, and hormones in the body in overweight PCOS patients.	Compared to Metformin alone, COM was more effective in increasing insulin sensitivity and decreasing anthropometric measures in overweight PCOS patients.	Study duration was short, small sample size, open-label design.
Gupta et al., 2016 [35]	Venezuela and USA	Randomized Controlled Trial	Pioglitazone: n = 16; Placebo: n = 16	Investigating the impact of Pioglitazone on DCI-IPG release, which is an insulin mediator.	Pioglitazone was shown to increase the release of insulin-stimulated DCI-IPG, which may lead to an increase in insulin sensitivity in patients with PCOS.	Unable to explain the mechanism of action.
Heidari et al., 2019 [36]	USA	Randomized Controlled Trial	Metformin: n = 29; No Treatment: n = 13	Study how metformin impacts endothelial dysfunction found in patients with PCOS.	In PCOS patients, regardless of changes in dyslipidemia, glucose metabolism, or prediabetes, Metformin was shown to improve endothelial function.	Short study duration (follow-up), no placebo control group, unknown whether endothelial cell dysfunction is specific to PCOS phenotype or genotype.
Tao et al., 2021 [37]	China	Randomized Controlled Trial	Exanatide: n = 50; Metformin: n = 50; Combination Therapy: n = 50	Test the clinical effectiveness of Metformin, Exenatide, or combination therapy in prediabetic women with PCOS.	Women taking Exenatideor combination therapy achieved higher rates of prediabetes remission compared to those only taking Metformin, by enhancing postprandial insulin secretion.	Specific population, open-label design.

*Vitamins and Supplements* 

Nineteen studies focused on treating the symptomatology of PCOS with the use of vitamins and supplements (Table 4), representing 46% of included studies (Figure 3). All studies showed a positive association between the tested vitamin/supplement and improvement in hormonal and/or metabolic dysfunction. Administration of a combination of Dextrin/Co-enzyme Q10/Omega-3s or Puerarin/Myo-inositol/Alpha-lactalbumin/Folic acid was shown to improve hirsutism by decreasing sex hormone production, LH:FHS ratio, testosterone, and androstenedione [38-41]. Specifically, resistant dextrin, a prebiotic that is mostly digested once it reaches the colon where it ferments, may improve the hirsutism and menstrual irregularities that are hallmark symptoms of PCOS [38]. Green cardamom supplemented with a low-calorie diet, symbiotic supplementation, and resveratrol was shown to significantly decrease inflammatory markers such as tumor necrosis factor-α (TNF-α), C-reactive protein (CRP), vascular endothelial growth factor (VEGF), and hypoxia-inducible factor (HIF)-1 [50-52]. A study on cinnamon supplementation and a study on spinach-derived thylakoids combined with a low-calorie diet both demonstrated a decrease in fasting blood glucose [46,47]. Three studies investigated vitamin D, which showed improvement in metabolic and hormonal parameters such as a decrease in plasma glucose, liver markers signaling injury and fibrosis, and total testosterone [42,48,49]. Vitamin D and selenium were each combined with a probiotic, and both resulted in improvements in mental health, as assessed by questionnaire, and metabolic parameters such as CRP levels, total glutathione, and total antioxidant capacity [43,44]. Vitamin K2 was associated with an increased QoL, based on improvement in questionnaires assessing depression and emotional health [55]. Magnesium alone was found to significantly impact QoL as determined by the Health Survey Quality of Life Questionnaire in women with PCOS by improving emotional well-being, energy level, and physical functioning [56]. 

**Table 4:** Studies on Vitamins and Supplements AMH = anti-Mullerian hormone; BMI = body mass index; CRP = C-reactive protein; FSH = follicle stimulating hormone; HOMA-IR = homeostatic model assessment for insulin resistance; LH = luteinizing hormone; NO = nitric oxide; PCOS = polycystic ovarian syndrome; QoL = quality of life

Reference	Region/Country	Study Design	Data Collection	Study Aim	Conclusion	Limitations
Gholizadeh et al., 2019 [38]	Iran	Randomized Controlled Trial	Prebiotic: n = 31; Placebo: n = 31	Determine how resistant dextrin can improve metabolic parameters in PCOS.	Resistant dextrin improves metabolic parameters and improves hirsutism and irregularities in menstrual cycles for women with PCOS.	Small sample size.
Hager et al., 2019 [39]	USA	Randomized Controlled Trial	“Profertile Female” + Unlabeled Tablet Containing Co-Enzyme Q10, Selenium, Vitamin E, Folic Acid, Catechin, and Glycyrrhizin: n = 30; Two Tablets With Only Folic Acid: n = 30	To evaluate whether co-enzyme Q10, omega-3s, and other antioxidants improve metabolic/endocrine parameters in PCOS patients.	The supplements taken consisted of folic acid, vitamin E, selenium, omega 3, catechin, co-enzyme Q10, and glycyrrhizin, which are effective at lowering LH:FSH, testosterone, AMH.	Small sample size, impact of each ingredient could not be assessed.
Li et al., 2021 [40]	China	Randomized Controlled Trial	Addition of Puerarin (Obese Treatment Group): n = 15; Addition of Puerarin (Non-Obese Treatment Group): n = 21; Standard Modality Treatment (Obese Control Group): n = 15	Investigate the impact of Puerarin (derived from a Chinese kudzu root) in patients with PCOS.	Puerarin improved sex hormones, oxidative stress, and other metabolic markers in both obese and non-obese groups compared to the standard modality treatment.	Small sample size, from the same area, could not rule out interaction with first-line medications.
Hernandez et al., 2021 [41]	Mexico and Italy	Non-Randomized Controlled Trial	Myo-Inositol + Alpha-Lactalbumin + Folic Acid: n = 34; Baseline Comparison of the Same Patients: n = 34	Investigating myo-inositol + alpha-lactalbumin on hormones and metabolic markers in PCOS patients.	Myo-inositol with alpha-lactalbumin improved insulin-resistance and sex hormone levels in different metabolic profiles of PCOS patients.	Small sample size, heterogeneous population (obese and non-obese).
Jamilian et al., 2017 [42]	Iran	Randomized Controlled trial	4000 IU Vitamin D: n = 30; 1000 IU Vitamin D: n = 30; Placebo: n = 30	Investigate Vitamin D supplements impact on insulin resistant PCOS patients, specifically its effect on their metabolic parameters.	Vitamin D supplement in a large dose (compared to smaller dose or placebo) showed more improvement on metabolic, endocrine, and inflammatory parameters.	Small sample size, insulin resistance eval based on HOMA-IR only, use of metformin could not be controlled.
Jamilian et al., 2018 [43]	Iran	Randomized Controlled Trial	Selenium + Probiotic: n = 30; Placebo: n = 30	Investigating how selenium with a probiotic impact on parameters of emotional well-being, inflammation, and hormones in PCOS patients.	Administration of selenium with a probiotic showed improvements on beneficial effects on emotional well-being, hormonal and inflammatory levels in PCOS patients.	Small sample size, circulating Selenium levels were not measured, no probiotic control group.
Ostadmohammadi et al., 2019 [44]	China	Randomized Controlled Trial	Vitamin D + Probiotic: n = 30; Placebo: n = 30	Probiotics with Vitamin D supplementation impact on emotional well-being, hormones, oxidative stress, and inflammation in PCOS patients.	Administration of vitamin D with probiotics shows improvements in emotional well-being, hormone levels, and inflammatory markers.	Small sample size, subjective measurement of emotional well-being, gut microbiome was not measured, other parameters of oxidative damage were not measured.
Haidari et al., 2020 [45]	Iran	Randomized Controlled Trial	Lifestyle Modification + Brown-Milled Flaxseed: n = 24; Lifestyle Modification: n=24	Investigate the impact of flaxseed with lifestyle vs. only flaxseed on weight and metabolic/inflammatory markers in PCOS.	Flaxseed with lifestyle modifications showed more improvements than just lifestyle alone in metabolic/inflammatory markers and weight in PCOS patients.	Small sample size, open-label design (no placebo).
Borzoi et al., 2018 [46]	Asia	Randomized Controlled Trial	Cinnamon: n = 42; Placebo: n = 42	Cinnamon impact on metabolic levels like serum glucose, lipids, and adiponectin in PCOS patients.	Cinnamon supplementation had improvement in some of PCOS patient’s metabolic parameters.	Short study duration, fixed treatment dose, specific sample population (BMI>25).
Tabrizi et al., 2020 [47]	Iran	Randomized Controlled Trial	Thylakoid-Rich Spinach Extract: n = 24; Placebo: n = 24	Investigation on the effect of spinach-derived thylakoids on metabolic parameters and obesity in PCOS patients.	Supplements of spinach-derived thylakoid vs caloric restriction alone showed increased insulin sensitivity and anthropometric indices in PCOS patients.	No follow-up for ovarian cysts, use of bioelectrical impedance to measure body composition, small sample size.
Javed et al., 2019 [48]	England	Randomized Controlled Trial	Vitamin D Supplement: n = 20; Placebo: n = 20	Assess how Vitamin D supplementation could affect the cardiovascular risk factors in women with PCOS.	The beneficial effects of vitamin D supplementation were on improving liver markers and insulin sensitivity in women with PCOS who vitamin D are deficient.	Excluded “gold standard” liver biopsy or other non-invasive methods as an additional approach to quantify liver fibrosis, relatively healthy population (no liver fibrosis).
Trummer et al., 2019 [49]	Austria	Randomized Controlled Trial	Vitamin D: n = 119; Placebo: n = 61	Studying if Vitamin D impacted metabolic/endocrine markers.	Vitamin D was shown to decrease plasma glucose during the oral glucose tolerance test. There was no effect on metabolic or endocrine markers with Vitamin D tablets in PCOS patients.	High drop-out rate, possible interaction of Vitamin D and insulin resistance, high baseline for concentration of serum 25(OH)D, Austrian monocentric study (results may not be generalizable).
Cheshmeh et al., 2022 [50]	Switzerland	Randomized Controlled Trial	Green Cardamom: n = 99; Placebo: n = 95	Investigate green cardamom’s effect on inflammatory markers in PCOS patients.	Green cardamom effectively reduces low-grade inflammation in PCOS patients.	Population too specific, gym closures and subject drop out due to COVID-19.
Bahramrezaie et al., 2019 [51]	Iran	Randomized Controlled Trial	Resveratrol: n = 30; Placebo: n = 31	To investigate Resveratrol’s impact on angiogenesis in patients with PCOS.	Resveratrol improved sex hormone levels and expression of granulosa cells angiogenesis’ markers.	Small sample size, wide variations in BMI may influence results.
Nasri et al., 2018 [52]	Iran	Randomized Controlled Trial	Synbiotic Supplementation: n = 30; Control: n = 30	Investigate the supplementation of synbiotic impact on inflammation, hormone, and oxidative stress markers in PCOS patients.	Synbiotic supplementation showed improvement on sex hormone binding globulin, hirsutism scores, hs-CRP, and NO levels, but not the other inflammatory biomarkers in PCOS patients.	Small sample size, short study duration, did not study other metabolic parameters, absence of including dose-response relationships.
Arentz et al., 2017 [53]	Australia	Randomized Controlled Trial	Herbal medicine + lifestyle intervention: n = 60; Lifestyle Intervention: n = 62	Investigate the impact of lifestyle changes + herbal medicine against lifestyle changes alone in overweight PCOS patients.	Lifestyle modification + herbal supplement improved menstrual parameters, weight, and QoL in overweight PCOS patients.	Lack of placebo group, did not control for placebo effect, subjective reporting of data.
Azizi-Kutenaee et al., 2022 [54]	Iran	Randomized Controlled Trial	Letrozole + Lactofem: n = 20; Letrozole Alone: n = 20	Investigate the impact of adding a probiotic to letrozole vs. letrozole alone in patients with PCOS based on infertility, body image, and sexual functioning.	Addition of a probiotic to letrozole may improve fertility, satisfaction in body image and sexual functioning in PCOS patients.	Small sample size, subjective data, sample is from OB/GYN clinic patients (possible bias).
Tarkesh et al., 2022 [55]	Iran	Randomized Controlled Trial	Vitamin K: n= 42; Control: n = 42	Investigating the impact of Vitamin K2 on depression in PCOS patients.	Depression in the K2 group significantly improved compared to the control group after 8 weeks of treatment with daily Vitamin K.	Lack of funding prevented the collection of serum levels (additional data) on osteocalcin, adiponectin and inflammatory markers.
Jaipur et al., 2022 [56]	Iran	Randomized Controlled Trial	Magnesium Supplement: n = 32; Placebo: n = 32	Impact of magnesium on abnormal uterine bleeding, QoL, acne, and alopecia.	Magnesium supplementation improved physical health, mental health, and fatigue. No significance was found on abnormal uterine bleeding, alopecia, or acne.	Small sample size due to COVID-19, some data was subjectively reported (recall bias).

The benefits of adding a supplement to lifestyle intervention were the focus of two other studies. One focused on flaxseed powder and lifestyle modification [45], while the other focused on herbal medicine and lifestyle modification [53]. These studies showed that certain supplements taken conjunctively with lifestyle modification resulted in a reduction in weight and insulin resistance [45,53]. Another study showed that adding a probiotic to letrozole therapy for the treatment of PCOS may decrease oxidative stress, leading to higher rates of pregnancy and improved sexual function [54]. 

*Alternative Interventions* 

Five studies, representing 12% of included studies (Figure 3), investigated alternative interventions to treating PCOS. These alternative, non-pharmacological interventions can be individualized to patients in treating their specific symptom profile versus the underlying pathology, which is targeted by pharmacological treatments (Table 5). The alternative interventions included in the review are cognitive behavioral therapy (CBT), osteopathic manipulative treatment (OMT), acupuncture, phlebotomy to reduce iron stores, and health education. Of these studies, phlebotomy had androgenic profile benefits such as regulating hyperandrogenism and insulin resistance [57], whereas acupuncture modifies sex hormone levels, reduces weight, and lowers anti-Müllerian hormone (AMH) [59]. One study concluded that weekly OMT can be useful in improving sympathetic tone, a component of the nervous system that responds to stress. Serum testosterone was also measured in these patients, and a significant decrease was seen in patients treated with osteopathic manipulation (OMT) [58]. One study surveyed patients before and after the implementation of an education model on PCOS and showed a significant improvement in their subjective nutrition and fitness status [60]. The last study used CBT and lifestyle modifications as their alternative interventions, which led to a significant average weight reduction in patients [61].

**Table 5:** Studies on Alternative Interventions AFC = antral follicle count; AMH = anti-Mullerian hormone; CBT = cognitive behavioral therapy; OCP = oral contraceptive pill; OMT = osteopathic manipulative treatment; QoL = quality of life

Reference	Region/Country	Study Design	Data Collection	Study Aim	Conclusion	Limitations
Behboudi-Gandevani et al., 2019 [57]	Iran	Randomized Controlled Trial	Phlebotomy: n = 32; Oral Contraceptive Pills: n = 32	Investigate the physiologic responses of women using phlebotomy compared to cyproterone acetate-containing OCPs to improve hyperandrogenemia, insulin resistance, and iron stores.	OCPs with phlebotomy had little effect on androgen levels and insulin resistance, while OCPs alone showed the most improvement in treating irregularities of the menstrual cycle. For serum triglyceride levels, phlebotomy had the least adverse effect.	Short duration, confounders were not assessed (i.e., lifestyle), high number of subjects lost to follow-up in phlebotomy group, small sample size.
Davis et al., 2020 [58]	USA	Randomized Controlled Trial	OMT Intervention: n = 14; No Intervention: n = 11	Investigate whether OMT improves sympathetic tone and metabolic effects in PCOS patients.	Weekly OMT showed a decrease in sympathetic tone and serum testosterone which suggests that it could be used as another tool along with other therapy for women with PCOS.	Sample population is too specific, lack of a sham treatment group, limited number of controls.
Leonhardt et al., 2015 [59]	Sweden	Randomized Controlled Trial	Acupuncture + Manual Low-Frequency Electrical Stimulation: n = 33; Physical Exercise: n = 34 No Intervention: n = 17	Study if physical activity or electro-acupuncture in women with PCOS impacts their serum AMH, volume of their ovary, or AFC.	Volume of the ovary and AMH was reduced with electro-acupuncture . Physical exercise had no effect on AFC, AMH, or ovarian volume.	Results based on secondary analysis, placebo effect not controlled, no MRI done at end of study.
Dashti et al., 2022 [60]	Malaysia	Randomized Controlled Trial	Structured Education: n = 34; Control Group: n = 35	Assess the effectiveness of providing health education on nutrition and fitness for women seeking treatment for PCOS.	The course provided on health education was an effective adjuvant treatment for PCOS patients.	Unable to obtain blood lab panels, ultrasounds were limited to women suspected of having PCOS during the initial eligibility process, and physical activity and nutrition knowledge, attitude and practice were self-reported.
Cooney et al., 2018 [61]	USA	Randomized Control Trial	Cognitive Behavioral Therapy + Lifestyle Modifications: n = 7; Lifestyle Modifications Only: n = 8	Investigate the outcomes of lifestyle modification vs. CBT + lifestyle modification in PCOS patients.	CBT with lifestyle modifications most improved QOL and weight loss in women with PCOS.	Small sample size, short study duration. Symptoms specific to PCOS not evaluated.

Discussion

This review has revealed that there is a wide variety of treatment options to consider for patients with PCOS, depending on their individual symptoms and treatment goals. These can include both pharmacologic and non-pharmacologic treatments. Treatments fell into one of four general categories: hormone-modulating drugs, diabetes drugs, vitamins and supplements, and alternative interventions, with varying levels of success treating the array of symptomatology present in PCOS patients. In general, many of the medications mentioned can be efficacious as primary treatments, while vitamins and supplements and the alternative interventions can be effective as adjuncts to medication therapy to maximize symptom control.

Hormone-Modulating Drugs

There are several hormone-regulating drugs available to treat the symptoms of PCOS that are related to the reproductive system, such as oligomenorrhea or irregular menstrual cycles and infertility due to ovulation dysfunction. Most of these medications change serum levels of estrogen, whether the drug itself contains estrogen (OCPs) or affects the way the body handles estrogen (Letrozole, GnRH). This can help regulate the brain-ovarian axis in these patients, which is important in controlling ovulation and fertility. However, the mechanism of GH therapy does not involve estrogen and instead acts directly on the ovary.

Medications that modify estrogen or its conversion to androgens are Letrozole and CC, commonly used to treat infertility in PCOS. Letrozole and CC are both aromatase inhibitors, preventing the conversion of estrogen to androgen. With more estrogen around to act on the brain-ovary axis, ovulation is more likely to occur. One study found Letrozole to be more effective at inducing primary ovulation over CC [25]. Fertility was also found to improve with adding human menopausal gonadotropin (HMG) to Letrozole and CC, especially in CC-resistant patients [28]. HMG contains LH and FSH and promotes follicle maturation in the ovary. Another treatment effective at increasing pregnancy rates is GnRH antagonists, such as Cetrotide. Since GnRH induces LH and FSH production in a cyclic manner, it must be administered in a pulsatile fashion to induce ovulation. GnRH antagonists have also been shown to decrease rates of OHSS, a serious complication of medication-induced ovulation [26].

OCPs are effective at treating the symptoms of PCOS related to hyperandrogenism and metabolic dysfunction and can even improve BMI. OCPs are commonly used in PCOS to correct the underlying hormonal imbalance and are often used with other methods such as lifestyle changes, behavior modification, and Metformin therapy. OCPs were found to decrease certain metabolic markers, such as adiponectin, a hormone produced by adipose tissue [22]. OCPs also affected lipid (HDL and LDL) levels, as well as lowered blood pressure - both common issues in PCOS patients [23,24]. Another imbalance found in PCOS is the LH to FSH ratio (LH:FSH), which is typically increased in patients with PCOS, resulting in ovulatory dysfunction. Fezolinetant, a non-hormonal drug used to treat hot flashes during menopause, was shown to decrease the ratio [21]. Having a decreased ratio is desirable as it can promote oocyte maturation and prevent early loss of pregnancy, as well as reduce hyperandrogenism seen in PCOS patients [15]. More evidence is needed to assess the impact of starting these interventions earlier in life and if it has any net effect on symptomatology. 

Finally, GH has been investigated as a drug that works directly on the ovarian follicle. GH is also being evaluated for its effects on reducing apoptosis of granulosa cells, the cells in the ovary that produce estrogen, in PCOS patients. Decreasing apoptosis can lead to higher quality oocytes and improved fertility due to the increased survival time of an ovarian follicle [27].

One advantage of OCPs and Letrozole is their relative affordability and availability, whereas newer drugs, such as Fezolinetant and GH, can be expensive for patients. Combinations of Letrozole and CC have proven to be effective in improving fertility, but the newer drugs mentioned have yet to be studied as combinations. As for OCPs, they are commonly combined with lifestyle modifications to address obesity and improve lipid profiles. They can be used in combination with other hormone-modulating drugs, but studies on the effectiveness of these are limited.

Diabetes Drugs

These medications are commonly used in patients with type 2 diabetes either to help the body produce its own insulin or improve responsiveness to insulin, lowering blood sugar over time. The most common glucose-lowering drug used for PCOS is Metformin, which has proven to be beneficial in treating prediabetic/diabetic pathology and obesity. Metformin acts to improve endothelial function in PCOS patients [36], preventing plaque formation and the development of cardiovascular disease. Further benefits for PCOS patients on Metformin include reduced acne, normalized hair growth patterns, and improvements in fertility, providing evidence of its antiandrogenic properties. The drug was even shown to improve patient’s subjective QoL [30], likely related to reduced symptomatology and improved functioning. Metformin, used in conjunction with Exenatide, reduced BMI and waist circumference in women who were overweight [34]. Metformin enhances insulin sensitivity, while Exenatide stimulates insulin production from the pancreas. The synergy between these mechanisms underlies their combined effectiveness. Another study showed increased rates of prediabetes remission in women with PCOS taking Metformin and Exenatide or Exenatide alone, when compared to Metformin alone [37], providing evidence for Exenatide’s superiority to Metformin for prediabetes. 

Liraglutide, a drug in the same class as Exenatide, also leads to weight loss [29] and lowers atherothrombotic markers in PCOS patients, reducing the risk of cardiovascular disease [32]. Other treatments, such as Pioglitazone and Sitagliptin, also used in type 2 diabetes, have different mechanisms of action but ultimately increase insulin sensitization in PCOS [33,35]. When Pioglitazone is complexed with Metformin (PM), patients reported decreased psychological stress. Researchers postulated this is due to lower serum testosterone levels and/or the drug’s inhibition of the NLRP3 Inflammasome, an enzyme involved in the inflammatory cascade [31]. On the other hand, Sitagliptin decreased visceral abdominal fat (VAT) and lowered maximum blood sugar reading during the oral glucose tolerance test (OGTT), both diagnostic measurements used to assess the risk for type 2 diabetes. Sitagliptin also lengthened the half-life of GH, which plays a role in reproductive health [33]. This aspect of Sitagliptin may provide an additional benefit for patients with PCOS, as it increases the length of time GH stays in the serum and, therefore, acts on ovarian follicles to promote ovulation. As insulin resistance is one of the most common consequences of PCOS, medications used to treat diabetes play an important role in treatment. By understanding the specific outcomes of each of these therapies, physicians are better equipped to tailor therapies to patients and subsequently brighten outcomes in PCOS symptomatology. 

Vitamins and Supplements

Supplemental therapies for PCOS include vitamins, herbs, and probiotics recommended to be used in combination with prescription medications, lifestyle changes, and/or alternative interventions to demonstrate effectiveness. The studies reviewed here show these supplements have a wide variety of benefits for patients experiencing symptoms associated with PCOS.

Many supplements address the systemic inflammation seen in PCOS [8]. Puerarin, a compound extracted from the kudzu root and commonly used in traditional Chinese medicine to treat pain, was shown to decrease serum inflammatory markers and oxidative stress. It also improved serum levels of sex hormones, both of which may be beneficial for patients with PCOS [40]. Vitamin D deficiency has been linked to PCOS, potentially contributing to insulin resistance and systemic inflammation [62]. Inflammatory markers were shown to decrease with the addition of vitamin D to daily probiotic therapy, likely due to the vitamin’s innate anti-inflammatory properties [50]. This may explain why vitamin D also improves liver markers for injury and fibrosis - an important benefit because non-alcoholic fatty liver disease is commonly found in women with PCOS [48]. Cardamom and flaxseed are dietary supplements that reduce inflammation. Cardamom was combined with a low-calorie diet [50], while flaxseed was combined with lifestyle changes [45] - both showing improved inflammation when compared to placebo. Selenium, when combined with a probiotic, significantly improved serum markers of inflammation [43]. Probiotics, as seen here, are commonly used with specific supplements in PCOS. One study looked at the effect of Synbiotics, a combination of prebiotics and probiotics, and found reduced nitric oxide (NO) and CRP, which are components of the inflammatory cascade [52]. When used in conjunction with various treatments, these substances have revealed their significant anti-inflammatory properties. The supplements mentioned in this section may also address oxidative stress, a consequence of chronic inflammation, although this was only specifically measured in the study on Puerarin.

Insulin resistance, another common symptom of PCOS, can be addressed with supplements such as spinach-derived thylakoids and cinnamon when combined with a low-calorie diet [46, 47]. Both showed a decrease in fasting blood glucose levels, a sign of improved insulin sensitivity in the body. It is thought that thylakoids can help decrease appetite, whereas cinnamon increases insulin production by the pancreas and slows gastric emptying, which are all mechanisms that work to lower blood sugar. Vitamin D supplementation has also shown improvements in insulin resistance, as demonstrated by lower blood sugars during an OGTT in patients taking vitamin D [49]. Two of these supplements (spinach-derived thylakoids and vitamin D) can also improve the hyperandrogenism of PCOS. Patients taking thylakoids showed decreased levels of testosterone, potentially providing benefits for those experiencing hirsutism and acne [47]. Hirsutism was also reduced by the combination of vitamin D and daily probiotics, in addition to its previously mentioned benefits [44]. Resistant dextrin, a soluble dietary fiber commonly used to lower post-prandial blood glucose and promote bowel movements, was shown to reduce testosterone production, which may help alleviate acne and hirsutism, as well as regulate the menstrual cycle [38]. 

Furthermore, a subset of supplements targeted hormones that regulate ovulation and, therefore, fertility. Reproductive abnormalities, such as LH:FSH imbalance and elevated testosterone levels, showed improvement with a trial of a daily tablet containing co-enzyme Q10, selenium, vitamin E, folic acid, catechin, and glycyrrhizin [39]. This outcome likely stems from a synergistic effect, and the effectiveness of each component was not evaluated individually. Resveratrol, a polyphenolic compound found in the skin and seeds of grapes and peanuts, was evaluated alone in a separate study. While it also improves hormone levels, its unique mechanism involves the growth of blood vessels in the ovary, improving blood flow to granulosa cells, which stimulates ovulation [51]. When myo-inositol, a native sugar, was used in combination with alpha-lactalbumin, a small protein found in dairy, patients saw reduced insulin resistance and an improved LH:FSH ratio [41]. The mechanism of alpha-lactalbumin is not well understood, but the increase in FSH production is due to myo-inositol acting as a cofactor. With a lower ratio, ovulation is more likely, and overall fertility may improve. There are many dietary supplements, herbs, and vitamins that address insulin resistance, many of which also happen to improve the sex hormone profile. With the dual mechanisms of supplements like thylakoids, vitamin D, resistant dextrin, and myo-inositol with lactalbumin, patients with PCOS can treat multiple symptoms with the same supplement.

QoL is commonly reported by PCOS patients to be decreased. Magnesium, as a daily supplement, was found to significantly impact QoL as determined by the Health Survey Quality of Life Questionnaire by improving emotional well-being, energy level, and physical functioning [56]. The mechanism for this is not well defined, although magnesium is thought to improve sleep and digestion, which may contribute to QoL. Patients with PCOS also commonly experience depression, a factor contributing to a decreased QoL. Vitamin K2, found in many fermented foods, organ meats, and dairy products, was shown to improve depression after eight weeks of daily supplementation [55]. It has further been found to be produced by gut bacteria, supporting evidence that probiotics can also contribute to improved mental health. The mechanism of vitamin K2 in the brain is not well known. Other components of QoL, like body image, fertility, and sexual functioning, were found to improve after the addition of a probiotic to Letrozole when compared to Letrozole alone [54]. This further adds to the benefits of probiotics in PCOS. Another study assessed the effects of herbal medicine, defined as a tablet containing *Cinnamomum verum*, *Glycyrrhiza glabra*, *Hypericum perforatum*, and *Paeonia lactiflora*, or a tablet containing *Tribulus terrestris*. Subjects taking either tablet showed improved menstruation, weight, and QoL when combined with lifestyle changes [53]. The way these supplements and herbs affect QoL is likely related to the alleviation of bothersome symptoms, leading to improved functioning. As depression and low QoL reporting are common among patients with PCOS, physicians should be aware that these over-the-counter supplements offer low-risk alternatives, particularly for patients leery of prescription medications.

While supplements are not typically used as a monotherapy to treat PCOS, including them in treatment protocols has potential benefits depending on individual patient physiology. While the use of supplements can cause apprehension among some physicians, their minimal side effect profile has made them a more popular option among patients in recent years. Doctors should be aware of any supplements their patients are taking to prevent potential medication interactions.

Alternative Interventions

Alternative interventions refer to therapies that address the whole patient and usually do not involve prescription medication. Various alternative interventions were found effective as adjunctive therapies in modulating the various symptoms of PCOS, such as acupuncture, phlebotomy, and CBT. Acupuncture treatment resulted in lower ovarian volume and AMH levels in PCOS patients, compared to exercise, which showed no effects in these parameters [59]. Ovarian volume is commonly increased in PCOS, partially due to high levels of AMH, which inhibits the development of follicles, turning them into large cysts. Lowering AMH can allow follicles to develop and decrease ovarian volume, thereby improving ovarian function.

Testosterone and insulin resistance can also be treated with an alternative intervention. Serum testosterone was found to be decreased in PCOS patients who were treated with weekly OMT [58], which may help patients with hirsutism and/or menstrual irregularities. Because OMT modulates autonomics, this effect on testosterone may involve a complex interplay between the sympathetic nervous system and the endocrine system. Phlebotomy was also assessed for its effects on hormones. However, when phlebotomy was compared to OCPs in reducing insulin resistance and testosterone levels, OCPs proved to be more effective. The only benefit found from phlebotomy was a reduction in triglyceride levels with a low rate of adverse effects [57]. With a lack of robust evidence to support widespread use, phlebotomy may be best saved for severe cases of hypertriglyceridemia.

It was found that some patients lack understanding of what it means to have PCOS and their treatment options. The outcomes of a structured health education program were the focus of one study, finding subjectively improved nutritional and fitness status of patients [60]. This evidence drives the importance for doctors to educate patients on PCOS and the impact that lifestyle changes can have on their symptoms. Another alternative option is CBT, which improved QoL scores when compared to lifestyle changes alone [61]. CBT has been proven to treat depression and anxiety, which can increase patients’ involvement in their own treatment plans and maximize their chances of success. It is important to note that PCOS may require multiple adjunct therapies, and an integrated approach has been shown to provide specific improvements, as discussed in this section.

QoL

It is common for patients with PCOS to have BMIs in the overweight and obese ranges. Studies demonstrate that this patient population has significantly higher levels of depression and anxiety, driven by higher body image distress (BID) scores [63]. Even in light of current treatments that relieve symptomatology, women with PCOS continue to report a decreased quality of life [64]. The reasons behind this will vary from patient to patient, with symptoms of infertility, hyperandrogenism, and high BMI presumed to contribute the most. Assessment of the gaps in traditional treatment may positively impact the trajectory of the current model of care by focusing more on the patient’s unique set of symptoms and preferences as well as exploring newer, less commonly known treatment options that traditional care plans may overlook. This can be accomplished with an integrated care plan [65].

Limitations

The most common limitation across all included studies is small sample sizes. Other limitations within the studies include short study duration, no measurements of symptomatology specifically in hormone drugs, restricted BMI in participants, and loss of follow-up. Limitations of this review that may have limited the number of potential search results include utilization of a small number of databases, not using additional synonymous search terms, and only including unrestricted access resources. However, the database search yielded 41 articles that met the search criteria using the PRISMA guidelines, and all met a high standard of quality as assessed by the JBI critical appraisal tools. When considering the studies on vitamins and supplements, many of the interventions were used in combination, making it difficult to attribute any positive effects to a singular substance.

Future Research

There are still various unknowns regarding the pathophysiology of PCOS. Future research is required to elucidate the genetic and/or environmental origins of PCOS and, subsequently, novel therapies targeting or reducing the primary pathogenesis. One of the main gaps in research is knowing which treatments are more efficacious when compared to others. Although some comparison studies were discussed in this paper, more are needed to assemble a complete picture of therapy options. Furthermore, additional research is needed to explore the time at which treatment is begun. For instance, which treatments should be started early to maximize their effects over time and which treatments should only be used for limited periods of time to minimize side effects.

## Conclusions

There is a wide range of treatment options with varying levels of efficacy for patients with PCOS that patients and health care providers should consider when treating symptoms. The treatment categories for PCOS can be broken up into hormonal therapy, diabetic medications, vitamins or supplements, and alternative interventions. As it stands, there is no first-line, single standard-of-care treatment or medication for all patients with PCOS, as symptomatology and treatment goals can vary greatly. OCPs are the most frequently used hormonal therapy for the menstrual irregularities of PCOS and are found to correct underlying hormone imbalance and improve metabolic parameters such as BMI. There are several adjunctive treatments that have similar effects, such as Puerarin, vitamin D, cinnamon, selenium, and flaxseed. These tend to work best when combined with standard care or lifestyle changes. When fertility is desired, Letrozole, Clomiphene, GH, and GnRH antagonists can help to induce ovulation. If these are ineffective, the addition of probiotics, myo-inositol, and herbal supplements has also demonstrated improvements in fertility. Many PCOS patients must also constantly battle with insulin resistance and type 2 diabetes. The most used diabetic therapy is Metformin, which improves insulin sensitivity to treat pre/diabetes in PCOS and increase quality of life. However, there are many other diabetic drugs with benefits for patients with PCOS, including Exenatide, Liraglutide, and Pioglitazone. In addition to improving insulin resistance, these drugs each have their unique benefits, such as decreased cardiovascular risk in the case of Liraglutide. There is evidence that Exenatide is just as, or more effective than, Metformin at improving metabolic parameters.

There are many options outside of the current standard medications that are equally effective and should be used in PCOS treatment plans. As adjuncts to PCOS treatment, vitamins, supplements, and alternative interventions have shown to improve symptoms that may not be addressed by standard hormonal and diabetic medications. As pharmacological treatments may not provide complete symptom relief or improve a patient's fertility status, vitamins, supplements, and alternative interventions can be added to help increase the chance of treatment success. Regimens can be symptomatically individualized based on patient goals to enhance the overall QoL of women affected by this syndrome, such as adding OMT to lower testosterone levels or CBT to address depression. Further research may help reveal the success of therapies when they are individualized and highlight the importance of specific symptom pattern recognition and early treatment.
